# The Interface between Osteoporosis and Atherosclerosis in Postmenopausal Women

**DOI:** 10.5935/abc.20180050

**Published:** 2018-03

**Authors:** Neuza H. M. Lopes

**Affiliations:** Unidade de Coronária Crônica - Instituto do Coração (InCor) - Faculdade de Medicina da Universidade de São Paulo, São Paulo, SP - Brazil

**Keywords:** Atherosclerosis / physiopathology, Osteoporosis / physiopathology, Bone Diseases, Metabolic, Women, Postmenopause

In recent years, the association between osteoporosis and atherosclerotic disease has been described, regardless of patients' age, and highlighted epidemiologic and pathophysiologic similarities between arterial wall calcification and osteogenesis.^1,2^ Cross-sectional, prospective studies have pointed out significant negative association between cardiovascular mortality and low bone mass, osteoporotic fractures, vascular calcification, extension of coronary artery disease and abdominal aortic injury.^3-5^ Concomitant occurrence of both diseases seems to result from common pathophysiologic and molecular mechanisms between these conditions. However, it is still controversial whether a low bone mass is caused by arterial calcification or vice-versa, or whether these conditions only have the same pathophysiological mechanism.

Risk factors for osteoporosis and atherosclerotic disease include estrogen, parathyroid hormone, homocysteine and vitamin K deficiency, lipid oxidation products, inflammatory process, vitamin D excess, molecular pathways involved in both bone and vascular mineralization, and similar mechanisms of calcification involving vascular and bone structures.^6^ Arterial calcification is found in more than 90% of atherosclerotic lesions. The process starts with formation of vesicles in the endothelial matrix, followed by proliferation and mineralization of the arterial intima-media wall, similarly to the bone tissue. Many bone remodeling regulators have been described in calcified atherosclerotic lesions, including osteocalcin, hydroxyapatite crystals, osteopontin, bone morphogenetic protein 2 osteoprotegerin, sclerostin, dickkopf factor (DKK), leptin, oxidized lipids and calcium sensor-related factor.^7^

Vascular atherosclerotic disease is more common in women with osteoporosis and osteopenia as compared with women without these conditions.^5,6^ Increased mortality rates related to cardiovascular diseases have been reported at advanced ages in postmenopausal women with low bone mineral density (BMD). Despite a non-significant increase in myocardial infarction among women with low BMD, with a rate of 22%, a significant increase is observed among men with low BMD, with a rate of 39%.^2^

In the present issue, the study by Cheng et al.^8^ adds to existing literature demonstrating an inverse association between BMD and coronary artery disease in postmenopausal women. The authors studied 122 postmenopausal women with diagnosis of coronary artery diseases (acute coronary syndrome or stable angina). All patients had undergone routine bone densitometry within one year prior to the assessment of atherosclerotic load by the Gensini score and invasive angiography. BMD of the femoral neck was measured by dual-energy X-ray absorptiometry. The presence of osteopenia/osteoporosis in femoral neck was associated with increased risk of severe coronary lesions. The multiple regression model revealed the T-scores as independent predictors of higher Gensini scores. This study corroborates previous data indicating an association between BMD and the severity of coronary atherosclerotic disease, suggesting that this parameter may be an independent marker of disease severity. 

Prospective studies including a larger number of patients and serial test data of BMD are needed to establish the role of T-scores as risk predictors for the development of severe coronary artery disease in men and women. Evidence from clinical practice suggests that osteoporosis patients should also be assessed for the risk of severe coronary artery disease.
